# Three Repeat Isoforms of Tau Inhibit Assembly of Four Repeat Tau Filaments

**DOI:** 10.1371/journal.pone.0010810

**Published:** 2010-05-25

**Authors:** Stephanie J. Adams, Michael A. DeTure, Melinda McBride, Dennis W. Dickson, Leonard Petrucelli

**Affiliations:** Department of Neuroscience, Mayo Clinic College of Medicine, Jacksonville, Florida, United States of America; Federal University of Rio de Janeiro, Brazil

## Abstract

Tauopathies are defined by assembly of the microtubule associated protein tau into filamentous tangles and classified by the predominant tau isoform within these aggregates. The major isoforms are determined by alternative mRNA splicing of exon 10 generating tau with three (3R) or four (4R) ∼32 amino acid imperfect repeats in the microtubule binding domain. In normal adult brains there is an approximately equimolar ratio of 3R and 4R tau which is altered by several disease-causing mutations in the tau gene. We hypothesized that when 4R and 3R tau isoforms are not at equimolar ratios aggregation is favored. Here we provide evidence for the first time that the combination of 3R and 4R tau isoforms results in less *in vitro* heparin induced polymerization than with 4R preparations alone. This effect was independent of reducing conditions and the presence of alternatively spliced exons 2 and 3 N-terminal inserts. The addition of even small amounts of 3R to 4R tau assembly reactions significantly decreased 4R assembly. Together these findings suggest that co-expression of 3R and 4R tau isoforms reduce tau filament assembly and that 3R tau isoforms inhibit 4R tau assembly. Expression of equimolar amounts of 3R and 4R tau in adult humans may be necessary to maintain proper neuronal microtubule dynamics and to prevent abnormal tau filament assembly. Importantly, these findings indicate that disruption of the normal equimolar 3R to 4R ratio may be sufficient to drive tau aggregation and that restoration of the tau isoform balance may have important therapeutic implications in tauopathies.

## Introduction

Tau filament assembly and deposition in the form of neuronal and glial fibrillary inclusions are the defining pathological features of a family of neurodegenerative diseases termed tauopathies [1]. Six predominant tau protein isoforms are generated in adult human brain by alternative splicing of the tau (*MAPT*) gene [2], [3], and each of the tau isoforms is likely to have a particular physiological role since they are differentially expressed during development [4], [5]. These isoforms differ by the presence or absence of two N-terminal inserts (exon 2 and/or 3 inclusion) and the presence of either three or four imperfect repeats (3R or 4R) in the microtubule binding domain at the C-terminus (exon 10 inclusion) [2], [6]–[8]. Some mutations in *MAPT* cause frontotemporal dementia and parkinsonism linked to chromosome 17 (FTDP-17) by disrupting the normal equimolar ratio of 3R and 4R tau isoforms [9]–[12]. Changes in the ratio of 3R and 4R tau do not appear to affect total tau expression significantly, suggesting that factors that alter exon 10 splicing may also contribute to other human neurodegenerative disorders, such as Pick's disease (3R inclusions) and progressive supranuclear palsy or corticobasal degeneration (4R inclusions). These findings suggest that neurodegeneration can be driven in humans regardless of direction of the shift in the normal equimolar 3R to 4R ratio [13], however, the mechanism by which these imbalances cause or contribute to the development of tau pathology and neurodegeneration remains unclear.

Alterations in the normal tau isoform ratio may not only affect tau function, given differences in 3R and 4R tau isoforms' ability to bind to and promote microtubule assembly [4], [14], but may also affect tau aggregation. Increased expression of exon 10 can alter the assembly properties of tau, as these amino acids reside in the core region of tau isolated from AD filaments [15]. Although some studies suggest that increased 4R tau expression may promote filament assembly under reducing conditions that may prevail in stressed neurons [16] [17], the effects of different tau isoforms ratios on tau assembly behavior has never before been reported. We hypothesize that the normal equimolar ratio of 3R and 4R tau isoforms, as observed in the adult human brain, does not favor aggregation; however, alterations in this equimolar ratio promote tau aggregation. Results presented here provide compelling evidence that altering the equimolar ratio of 3R and 4R tau such that 4R tau predominates accelerates tau filament assembly and that even small amounts of 3R tau can significantly inhibit the 4R tau assembly. Together these findings provide a framework for understanding how decreasing the normal 3R:4R tau ratio, present in many tauopathies, may drive tau aggregation and pathology. These results further suggest that therapeutic interventions aimed at returning the tau isoform ratios to their normal balance may benefit tauopathy patients.

## Materials and Methods

### Recombinant tau protein

All tau cDNAs in pET30a were expressed in competent BL21 (DE3) cells after growing to an OD (600) of 0.5 with 0.5 mM IPTG induction for 2.5 hours. Cell pellets were collected, washed and stored at −80°C before lysis with three freeze and thaw cycles. Tau proteins were then purified from inclusion bodies using high heat and ion exchange chromatography. These samples were further purified by semi-preparative HPLC C8 reverse phase chromatography if degraded fragments were detected in the original preparation [18]. Purity of the tau preparations was analyzed by SDS-polyacrylamide gel electrophoresis and Coomassie blue staining, and protein concentrations were determined using the BCA protein assay kit (Pierce, Rockford, IL).

### Tau assembly reactions

Tau protein in 10 mM HEPES (pH 7.4), 100 mM NaCl, 0.25 mM Phenyl-methane-sulfonyl fluoride (Sigma, St. Louis, MO) was incubated at 37°C in the presence of heparin (Sigma: MW 6 kDa) to induce assembly, and these reactions were carried out with 0–1 mM dithiothreitol (Acros Organics, Belgium) to examine the role of disulfide bond formation in the reactions. Sample reactions of 240–400 µl were incubated for 0–6 days. For the mixing experiments, molar ratios of 3R:4R were used in polymerization reactions to adjust for differences in the molecular weights of the tau isoforms. The reactions were performed with 8 µM total tau and 0.04 mg/ml heparin with the amounts of the two tau isoforms varying in 2 µM increments so that ratios of 1∶3, 2∶2 and 3∶1 were achieved in addition to the single isoform reactions at 8 µM tau that were used as positive controls. In all of the mixing reactions the molar ratio of tau and heparin was held constant. For the spiking reactions, the concentration of one isoform was fixed at 8 µM and the reaction was spiked with 0 µM, 1 µM, 2 µM, or 3 µM aliquots of a second isoform or buffer while the heparin amount was fixed at 0.4 mg/ml. Thus, in these reactions the molar ratio of tau and heparin was allowed to vary as the reactions were spiked with additional tau. Controls were also run at constant total tau and heparin ratios. Again these samples were incubated at 37°C for 0–6 days before assaying for tau filament assembly.

### Thioflavin S binding to tau

Tau intermediate and filament assembly were monitored by fluorescence of thioflavin S binding using a Cary Varian Eclipse Spectrofluorometer (Walnut Creek, CA) with an excitation wavelength of 440 nm with a slit width 10 nm, and emission spectrum collected from 460–600 nm again with a 10 nm slit width. Measurements were performed at room temperature after incubating 45 µl of assembly reaction with 45 µl of 0.006 mg/ml thioflavin S for 30 minutes. Negative controls included buffer alone, heparin and buffer, and reactions containing tau only with no heparin, but each with thioflavin S as above. Thioflavin S binding intensity was measured by integrating the curve between the ranges of 470–600 nm using the Cary Eclipse Scan software.

### Ultracentrifugation of aggregated tau

Samples from assembly reactions were centrifuged at 100 000×g for 75 minutes at 4°C to separate free and aggregated tau. Supernatants containing soluble tau were split in half for protein analysis by BCA and for SDS-PAGE by combining with 2× sample buffer. The pellets containing aggregated and polymerized tau were resuspended in 25 µl 1× sample buffer. Supernatants and pellets were visualized by SDS-PAGE on 10% Tris-glycine gels (Novagen) after Coomassie blue staining. For quantitation the gels of the pellets were scanned, and the bands quantified using Image J freeware [19]. Samples were normalized to the 4R tau values.

### Electron microscopy of filamentous tau

Reaction samples were diluted 4× in 10 mM HEPES, and 10 µl was adsorbed onto carbon/formvar-coated 400 mesh copper grids (EM Sciences) for 30 seconds. These were then stained with 2% uranyl acetate for 30 seconds, and the grids were examined with a Philips 208S electron microscope (Philips, Hillsboro, OR). For quantification, 6–8 images were collected randomly at 10,000× magnification, and the average filament number and total filament length per field was measured using Image J freeware [19].

### Statistics

Statistical analysis was performed using SigmaPlot 11.0 (San Jose, CA). Data are expressed as mean ± standard error of the mean (SEM). Statistical analysis was performed by ANOVA and all pairwise multiple comparisons procedures by the Student-Newman-Keuls Method, Spearman Rank Order Correlation, or t tests where appropriate, with p values of <0.05 considered significant. Graphing was performed using Graphpad Prism software (Graphpad, San Diego, CA). For gel quantification and spiking reactions, each sample was normalized to 4R tau, and the data is presented as mean relative fluorescence or mean relative tau aggregation ± SEM.

## Results

### Purification of Recombinant Tau Isoforms

**Figure 1:**
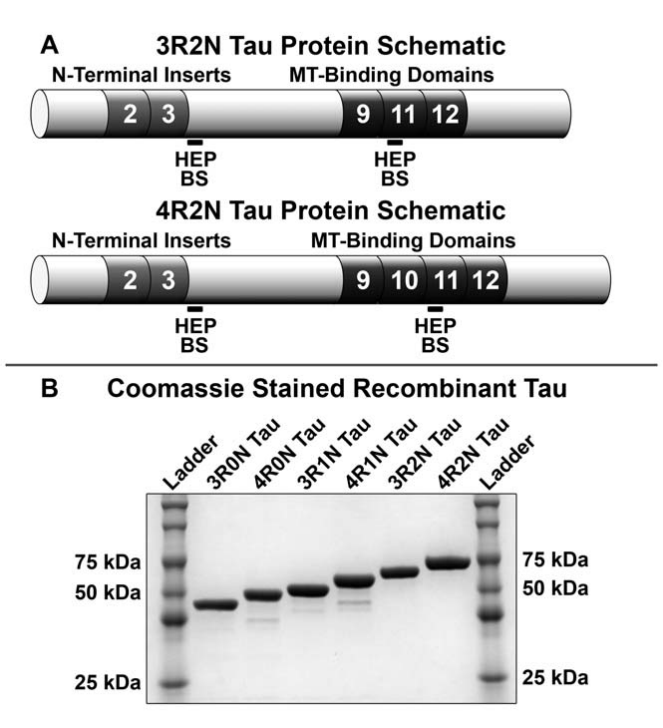
Schematic representation of human tau and Coomassie blue-stained gel of recombinant tau. (A) Full-length 3-repeat tau (3R2N) and 4-repeat tau (4R2N) containing N-terminal inserts encoded by exons 2 and 3, and microtubule (MT)-binding repeats encoded by exons 9–12. The 6 different tau isoforms are generated by alternative splicing of exons 2, 3, and 10 shown in white. The tau isoforms 3R0N and 4R0N would not include exons 2 and 3. Exclusion of alternatively spliced exon 10 generates 3-repeat tau isoforms. Inclusion or exclusion of alternatively spliced exons 2 and 3 generates 3R1N, 4R1N, 3R2N, or 4R2N tau isoforms. Potential heparin binding sites (HEPBS) are indicated by bars. (B) Purified recombinant tau proteins used in the study were loaded heavy at 2 ug per well and separated on 10% SDS-PAGE gels before staining with Coomassie brilliant blue R-250 to demonstrate purity.

Tau isoforms can be distinguished by the presence of 3 or 4 repeats in the microtubule binding domain in the C-terminus and by the presence or absence of 1 or 2 N-terminal inserts. Inclusion of alternatively spliced exon 10 leads to the generation of 4R tau isoforms, whereas exclusion of exon 10 leads to generation of 3R tau isoforms. Tau isoforms with 0, 1, or 2 N-terminal inserts are generated by alternative splicing of exons 2 and 3. A schematic representation of tau isoforms, 3R2N and 4R2N, including the microtubule binding repeat domains encoded by exons 9–12, the N-terminal inserts encoded by exons 2 and 3, and the location of potential heparin binding sites, are shown in Figure 1A. All studies were performed using highly purified recombinant tau preparations as shown in Figure 1B, and polymeric tau assembly, using heparin as an inducer, was measured using thioflavin S binding fluorescence.

### Assembly of Recombinant Tau into Filaments

**Figure 2:**
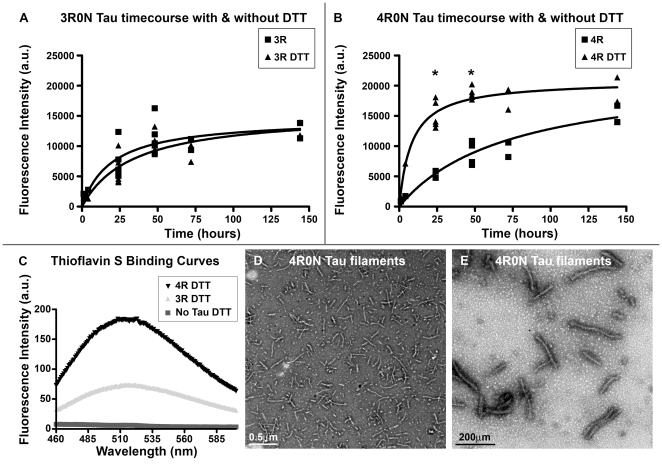
Tau Isoform Assembly Kinetics. The kinetics of 3R (A) and 4R (B) tau isoform assembly was measured using thioflavin S binding fluorescence in the presence of 0.04 mg/ml heparin with or without DTT. Each time point represents 2–5 experiments performed on separate days with background fluorescence (no tau present in reaction) subtracted from each experiment. (C) The thioflavin S data presented in A and B is the integrated value from thioflavin S binding curves as shown for 4R, 3R, and no tau reactions in the presence of DTT at day 1. (D–E) Electron micrographs of 4R0N tau assembly reactions containing 0.04 mg/ml heparin with DTT can be used to confirm the presence of tau filaments.

Assembly kinetics of 3R and 4R tau isoforms were assessed individually in the presence and absence of the reducing agent DTT. This data was collected as described in the methods section where thioflavin S fluorescence as shown in Figure 2C is integrated to measure tau progression into an assembly competent intermediate or polymer that is also observed in filamentous tau lesions in tauopathies [20], [21]. The 3R tau isoforms assembled similarly in reducing and non-reducing conditions (Figure 2A) whereas the 4R tau isoforms assembled more rapidly and reached a similar equilibrium value in the presence of DTT compared with 4R tau reactions in the absence of DTT (Figure 2B). Moreover, the assembly rate and the equilibrium value were greater than for 3R tau with or without DTT (Figure 2A and C). This accelerated 4R tau assembly observed under reducing conditions is in agreement with several previously published studies showing that 4R tau preferentially forms compact monomers, probably due to intramolecular disulfide bonds formed under oxidizing conditions. These compact monomers do not aggregate efficiently and may actually inhibit tau aggregation. In contrast under reducing conditions, 4R tau isoforms more rapidly assembled in the presence of polyanions [16] [17]. These data also demonstrate that steady state is reached by 48 hours under the reducing conditions tested here, and direct visualization with electron microscopy confirms the presence of tau filaments in both the 3R (data not shown) and 4R (Figure 2D–E) assembly reactions.

### Mixing 3R and 4R Tau Decreases Thioflavin S Binding

**Figure 3:**
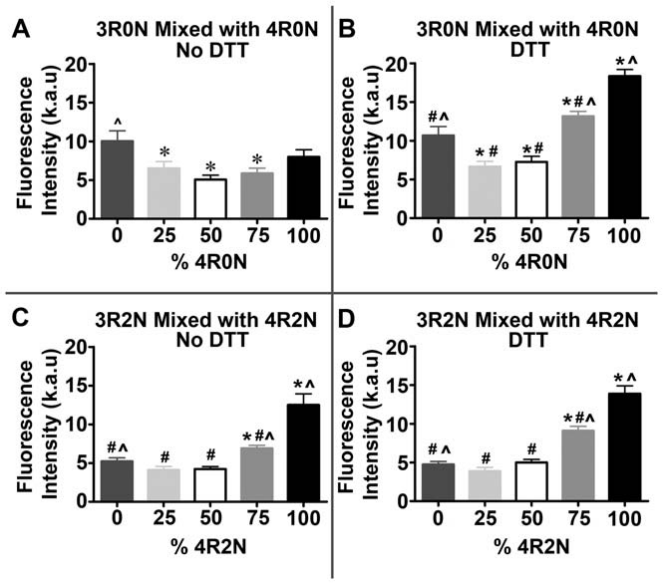
Ratio of 3R to 4R tau isoforms determines the extent of tau assembly. Thioflavin S binding fluorescence of different ratios of 3R and 4R tau isoforms in the presence of 0.04 mg/ml heparin with or without DTT was measured at 48 hours or steady state. 3R0N tau isoforms mixed with increasing molar fractions of 4R0N tau under non-reducing (A) and reducing (B) conditions. Ratios of 3R0N to 4R0N were as follows: 8 µM 3R0N (0% 4R0N), 6 µM 3R0N to 2 µM 4R0N (25% 4R0N), 4 µM 3R0N to 4 µM 4R0N (50% 4R0N), 2 µM 3R0N to 6 µM 4R0N (75% 4R0N) and 8 µM 4R0N (100% 4R0N). 3R2N tau isoforms mixed with increasing molar fractions of 4R2N tau under non-reducing (C) and reducing (D) conditions. Ratios of 3R2N to 4R2N were the same as for 3R0N to 4R0N. Statistical analysis was performed using a one way ANOVA (for 30/40 mixes with and without DTT and for 32/42 mixes with DTT) or by Kruskal-Wallis One Way Analysis of Variance on Ranks (for 32/42 without DTT) and all pairwise multiple comparisons were done by the Student-Newman- Keuls Method, with p<0.05 considered significant. An * denotes significance relative to 3R tau assembly, # denotes significance relative to 4R tau assembly, and ∧ denotes significance relative to the equimolar ratio of 4R:3R tau assembly.

To characterize the assembly behavior of different ratios of 3R and 4R tau isoforms, the assembly of tau isoform mixtures was compared with that of individual isoforms in the presence or absence of DTT. Mixtures of different molar ratios of 3R and 4R tau isoform do not assemble to the same extent as reactions containing single tau isoforms, and this is independent of the reducing conditions (Figure 3). The pure 3R tau isoform reactions assembled similarly in the presence or absence of DTT whereas the pure 4R reactions assembled better with DTT. Although 3R0N tau isoforms appeared to assemble better than 4R0N tau in the absence of DTT, the difference was not statistically significant (Figure 3A). Despite these relatively similar steady state levels of assembly for 3R0N and 4R0N tau isoforms individually under non-reducing conditions, mixing equimolar amounts of these 3R and 4R tau isoforms resulted in a significant decrease in tau assembly relative to 3R or 4R tau alone (Figure 3A). This reduction in tau assembly at the equimolar tau isoform ratio was also observed and accentuated under reducing conditions (Figure 3B). Here, shifting the ratio towards either pure 3R0N tau isoforms or 4R0N tau isoforms from the equimolar ratio increased total tau assembly significantly with the pure 4R0N isoforms assembling more than two-fold higher than the equimolar reactions (Figure 3B). Together, these data show that the equimolar ratio of 3R0N and 4R0N tau isoforms found in normal adult human brain is least favorable for aggregation, while shifting the ratio towards either 3R or 4R increases tau assembly. In addition, shifting the tau isoform ratio towards 4R tau, as occurs in certain tauopathies, especially under reducing conditions found in the cytoplasm of a cell, showed the greatest increase in tau assembly.

The presence of the N-terminal inserts did affect the assembly properties of the individual isoforms but the inhibition in assembly of the equimolar 3R and 4R reactions was still observed compared to the reduced single isoform values (Figure 3C–D). Three-repeat tau assembly was negatively impacted by the presence of the N-terminal inserts, independent of reducing conditions (Figure 3A–D). In contrast, 4R tau assembly was increased by the presence of the N-terminal inserts under non-reducing conditions (Fig. 3A vs 3C), but decreased by their presence under reducing conditions (Fig. 3B vs 3D). Despite these observed effects on 3R and 4R tau isoform assembly individually, the N-terminal inserts did not significantly affect the assembly of the equimolar ratio of 3R and 4R tau isoforms. Most importantly however, mixing equimolar amounts of 3R2N and 4R2N significantly reduced tau assembly relative to either pure 3R2N or 4R2N assembly reactions, and this was observed independent of reducing conditions (Figure 3C–D). Similar results were also obtained from experiments using tau 3R1N and 4R1N isoforms with one N-terminal insert (data not shown). Taken together, these data demonstrate that although the N-terminal inserts do affect individual tau isoform assembly, the primary determining factor on the extent of tau assembly is the ratio of 3R to 4R tau isoforms. This was further confirmed in mixing experiments with 4R0N and 4R2N that did not show depressed assembly levels under the equimolar conditions compared to the single isoform reactions (data not shown).

### Mixing 3R and 4R Isoforms Reduces Tau Aggregation

**Figure 4:**
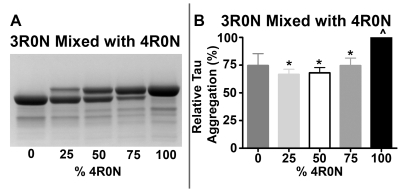
Tau aggregation decreased in mixed isoform reactions. Assembly reactions of single tau isoforms and different ratios of 3R0N/4R0N tau isoforms were centrifuged at 100,000×g to separate free from aggregated or polymerized tau. Coomassie stained gels of the pellets containing polymerized tau from A) 3R0N mixed with 4R0N with the following samples for each gel: 8 µM 3R0N (0% 4R0N), 6 µM 3R0N to 2 µM 4R0N (25% 4R0N), 4 µM 3R0N to 4 µM 4R0N (50% 4R0N), 2 µM 3R0N to 6 µM 4R0N (75% 4R0N) and 8 µM 4R0N (100% 4R0N). Densitometric analysis of pelleting gels B) 3R0N mixed with 4R0N was performed using Image J software [19]. Results were normalized to 4R values and relative % tau aggregation is shown. Statistical analysis was performed using a one way ANOVA and all pairwise multiple comparisons were done by the Student-Newman-Keuls Method, with p<0.05 considered significant. An * denotes significance relative to 4R tau assembly and a ∧ denotes significance relative to the equimolar ratio of 4R:3R tau assembly.

To establish that the tau assembly observed by thioflavin S binding fluorescence was due to tau aggregation and not simply an unstable tau intermediate without filament assembly, ultracentrifugation of assembly reactions was performed to separate soluble from aggregated or polymerized tau. Coomassie stained gels of the pellets containing polymerized tau showed that assembly reactions of 3R0N and 4R0N tau isoform mixtures again contained less aggregated tau than the single isoform reactions as observed under the reducing conditions of the cell. As shown in Figure 4A–B, 4R0N assembles to a statistically greater extent than equimolar 3R and 4R reactions whereas the 3R0N increase observed with thioflavin S could not be demonstrated statistically. These results for increased 4R tau were in agreement with the previous tau folding data in Figure 3, and they confirmed that the observed decrease in thioflavin S binding fluorescence in the mixed isoform reactions was due to a decrease in tau aggregation. Interestingly, some thioflavin S fluorescence was observed in the supernatants after high speed ultracentrifugation (data not shown) suggesting that thioflavin S fluorescence and tau folding intermediates precede aggregation, as previously described [21]. Similar results were observed in the 3R1N/4R1N and 3R2N/4R2N reactions (data not shown).

### Spiking 4R Tau Assembly Reactions with 3R Tau Inhibits Tau Assembly

**Figure 5:**
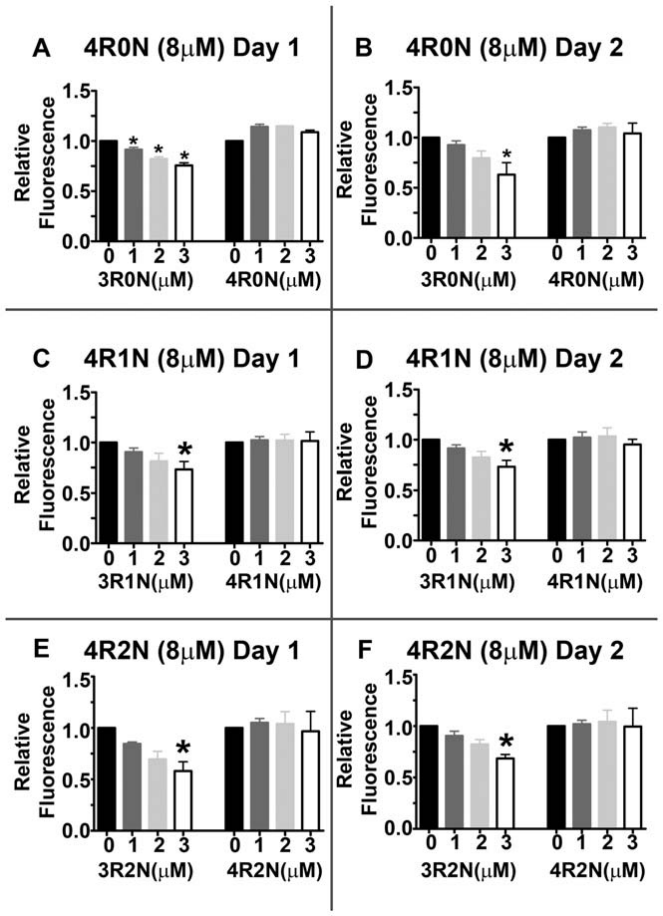
3R Tau Inhibits 4R Tau Assembly. Increasing molar concentrations (0, 1, 2, or 3 µM) of 3R or 4R tau were added to 8 µM 4R tau assembly reactions containing 0.04 mg/ml heparin and DTT, and Thioflavin S binding fluorescence was measured after 24 and 48 hours. 3R0N or 4R0N tau spiked into 4R0N tau reactions after 24 hours (A) and 48 hours (B). 3R1N and 4R1N tau spiked into 4R1N tau reactions after 24 hours (C) and 48 hours (D). 3R2N and 4R2N tau spiked into 4R2N tau reactions after 24 hours (E) and 48 hours (F). Fluorescence was normalized to 4R0N (A and B), 4R1N (C and D), and 4R2N (E and F) assembly and the relative fluorescence is shown. Statistical analysis was performed using a one way ANOVA and all pairwise multiple comparisons were done by the Student-Newman- Keuls Method, with p<0.05 considered significant. An * denotes significance relative to unspiked 4R tau assembly reactions. Addition of 3R tau isoforms to 4R tau assembly reactions showed a dose dependent inhibition of tau assembly, in spite of an overall increase in tau protein levels, relative to unspiked 4R tau assembly reactions (p = 0.018, p = 0.001, and p = 0.001 for 1 µM, 2 µM, and 3 µM 3R0N, respectively, for day 1 and p = 0.046 and p = 0.026 for addition of 2 µM and 3 µM 3R0N, respectively, for day 2). Additionally, 4R1N and 4R2N tau assembly reactions spiked with 3R1N and 3R2N tau isoforms, respectively, showed negative correlations between 4R tau assembly and additions of increasing concentrations of 3R tau isoforms (p<0.05 for both 4R1N and 4R2N) using the Spearman Rank Order Correlation test in Sigmaplot. Assembly of 4R tau reactions were either unchanged or showed a dose dependent increase in tau assembly following addition of more 4R tau isoforms.

Results from the mixing experiments suggested that even small proportions of 3R tau may inhibit the assembly of the 4R tau isoform. To test this more directly, tau assembly reactions containing different 8 µM 4R tau isoforms were spiked with increasing concentrations of 3R or 4R tau isoforms, and assembly was measured using thioflavin S fluorescence as before. Addition of 3R0N isoforms to 4R0N tau assembly reactions led to a dose dependent reduction in tau assembly (Figure 5A–B) again under reducing conditions, though reactions without DTT gave similar results (data not shown). Although these reactions contained more total tau protein, the addition of the 3R0N isoforms to the 4R0N assembly reactions resulted in less tau assembly than reactions of 4R tau alone containing less total tau protein. This inhibition of tau assembly appeared to involve a specific interaction between 3R and 4R tau isoforms, as addition of more 4R0N isoforms to 4R0N tau assembly reactions did not decrease assembly and appeared to modestly increase it (Figure 5A–B). Furthermore, as with the mixing experiments, N-terminal inserts did not block the inhibition of 4R assembly by 3R isoforms (Figure 5C–F). Since tau assembly reactions are extremely sensitive to changes in the tau to inducer ratio [21], these experiments were repeated by spiking the different 4R tau assembly reactions with either 3R or 4R tau isoforms plus heparin to maintain a constant tau to heparin ratio. Under these conditions, again the 4R spiked with 3R tau reactions displayed a dose dependent decrease in assembly while 4R tau spiked with additional 4R tau showed a dose dependent increase in tau assembly (data not shown). These data demonstrate that even small amounts 3R tau isoforms inhibit 4R tau assembly under reducing or non-reducing conditions.

### Spiking 4R Tau Assembly Reactions with 3R Tau Inhibits Tau Aggregation

**Figure 6:**
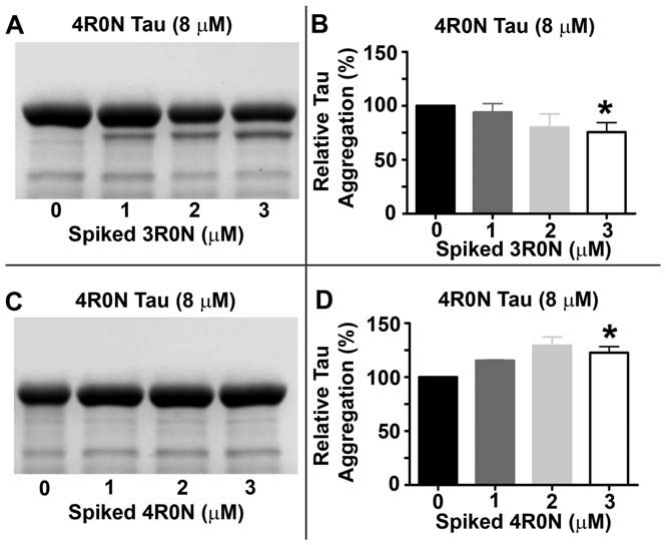
3R tau isoforms inhibit 4R tau aggregation. Different 4R tau isoform assembly reactions alone or spiked with increasing molar concentrations of 3R tau isoforms, with or without N-terminal inserts, were centrifuged at 100,000×g to separate free and polymerized tau. Coomassie stained gels of the pellets containing polymerized tau from A) 8 µM 4R0N spiked with 3R0N, C) 8 µM 4R0N spiked with 4R0N, with the following samples for each gel: 4R (0 µM 3R), 4R spiked with 1 µM 3R, 4R spiked with 2 µM 3R, and 4R spiked with 3 µM 3R. Densitometric analysis of pelleting gels B) 4R0N spiked with 3R0N and D) 4R0N spiked with 4R0N, was performed using Image J software [19]. Statistical analysis performed using the Spearman Rank Correlation test in Sigmaplot showed a negative correlation between 4R tau assembly and additions of increasing concentrations of 3R tau isoforms (p<0.05), but a positive correlation between 4R tau assembly and additions of increasing concentrations of 4R tau isoforms (p = 0.05).

As with the previous mixing experiments, confirmation that the decrease in thioflavin S binding fluorescence represented a decrease in tau aggregation in the assembly reactions of 4R tau spiked with 3R tau isoforms was obtained by ultracentrifugation. Coomassie stained gels of the pellets containing polymerized tau showed that assembly reactions of 4R tau spiked with 3R tau isoforms, despite containing more total tau protein, contained less polymerized total tau than 4R tau reactions alone (Figure 6A–B). In contrast, pellets from 4R tau assembly reactions spiked with more 4R tau isoforms contained more polymerized tau than 4R tau assembly reactions alone (Figure 6C–D). This is as expected, given that these reactions contain more total tau protein. In agreement with the thioflavin S binding fluorescence data, the N-terminal inserts had little effect on the inhibition of 4R tau aggregation by 3R tau isoforms. Polymerized tau in the pellets from 4R1N or 4R2N tau assembly reactions spiked with 3R1N or 3R2N, respectively, was either decreased or unchanged, despite the presence of more total tau protein in these reactions, relative to 4R1N or 4R2N tau reactions alone (data not shown).

### 3R Tau Inhibits the Nucleation and Extent of 4R Tau Filament Assembly

**Figure 7:**
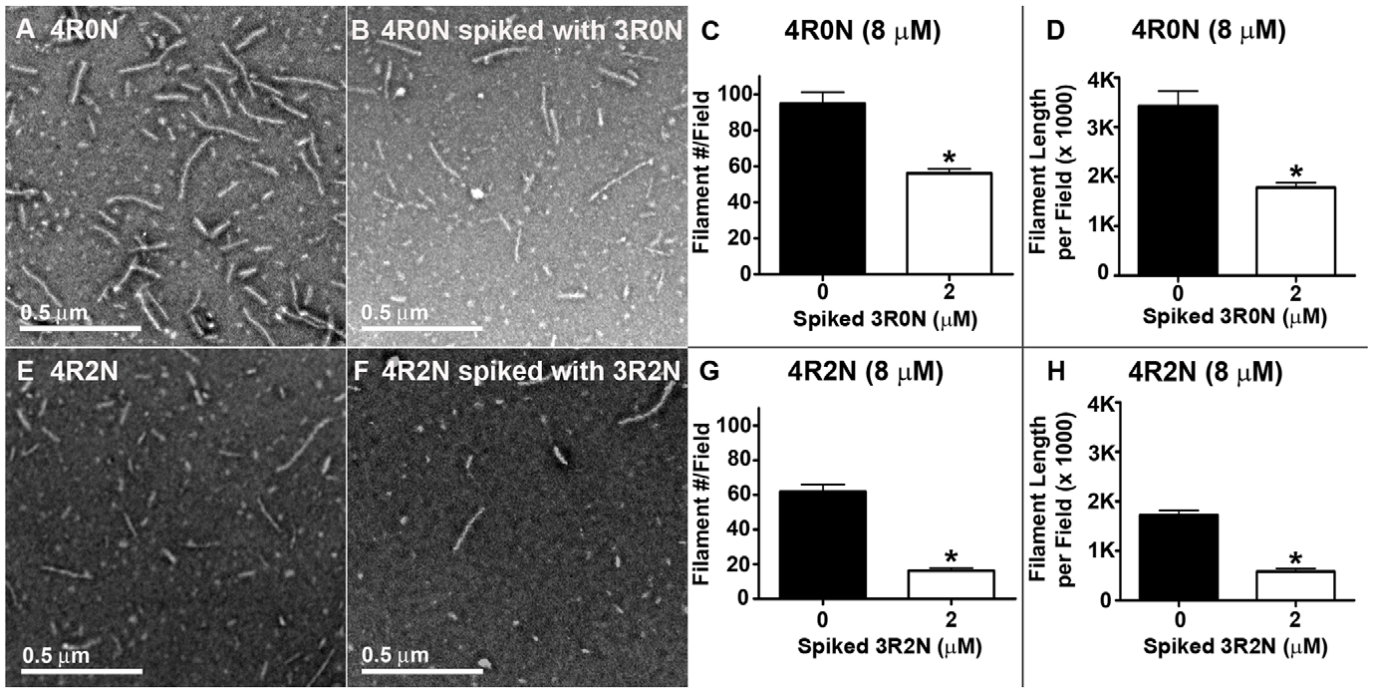
3R tau isoforms inhibit 4R tau filament formation. A) Electron micrograph of tau assembly reaction containing 8 µM 4R0N, B) Electron micrograph of tau assembly reaction containing 8 µM 4R0N spiked with 2 µM 3R0N, C–D) Quantification of average tau filament number and average tau filament length per field was performed on 6–8 images, collected randomly at 10,000× magnification, using Image J freeware [19]. E) Electron micrograph of tau assembly reaction containing 8 µM 4R2N, F) Electron micrograph of tau assembly reaction containing 8 µM 4R2N spiked with 2 µM 3R2N, and G–H) Quantification of tau filament number per field performed as above. Statistical analysis performed by t-test in SigmaPlot showed a significant decrease (p<0.0001) in tau filament formation and tau filament length (p<0.0001) in both 4R spiked with 3R and 4R2N spiked with 3R2N assembly reactions compared with unspiked 4R or unspiked 4R2N tau assembly reactions, despite an overall increase in total tau protein.

The effect of 3R tau isoforms on 4R tau isoform filament formation was also examined by electron microscopy. Electron micrographs established that the inhibition of 4R0N tau assembly and aggregation by 3R0N tau isoforms evident by decreased thioflavin S binding fluorescence and decreased tau aggregation was due to a decrease in tau filament formation in these reactions (Figure 7A–B). The number of tau filaments per field was significantly reduced in 4R0N tau assembly reactions spiked with 3R0N tau isoforms, again despite these reactions containing more total tau protein (Figure 7C). These results imply that seeding efficiency is decreased when 3R tau isoforms are added to 4R tau assembly reactions. The total length of tau filaments per field was also significantly decreased in the 4R0N tau assembly reactions spiked with 3R0N tau confirming the aggregation and thioflavin S data (Figure 7D). This decrease in the number and summed length of tau filaments following spiking of 4R tau assembly reactions with 3R tau isoforms occurred regardless of the presence of the N-terminal inserts (Figure 7E–H). The centrifugation assays and electron micrographs confirmed our thioflavin S binding fluorescence data by showing that even small amounts of 3R tau isoforms inhibit tau aggregation and more specifically tau filament formation. Since the N-terminal inserts did not interfere with this inhibition of 4R tau aggregation and filament formation by 3R tau isoforms, this data also confirms the decrease in tau filament formation is due to a specific effect depending on the microtubule binding repeats of the 3R and 4R tau isoforms.

### Tau Filament Morphology is Altered by the Presence of 3R and 4R Tau

**Figure 8:**
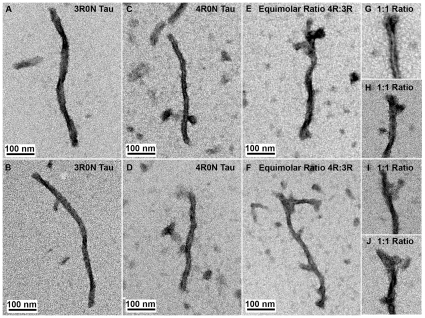
Changes in tau filament morphology in mixed isoform reactions. Filaments from 48 hour single isoform or mixed isoform assembly reactions were examined by electron microscopy. Representative filaments from reactions containing only 3R0N tau were observed as A) loosely twisted paired helical filaments or B) straight filaments while those formed in single 4R0N reactions contained predominantly C–D) straight filaments. The filament ends in these pure reactions were mostly observed to be well defined and cleanly stained. Filaments from the mixed isoform reactions were also able to form E) loose paired helical filament and F) straight filaments though the filament ends G–J) were often not well defined and demonstrated splaying or amorphous aggregation. The scale bar is 100 nm.

The dramatic decrease in filament number and total filament length per field observed by electron microscopy when either 4R0N or 4R2N tau was spiked with the corresponding 3R homologue suggests that 3R and 4R tau may not be exchangeable subunits for filament assembly. Although the work presented here clearly establishes that reaction mixtures containing single tau isoforms assemble to a greater extent than those containing both isoforms, the data does not provide any insight as to whether the filaments that do form when both 3R and 4R isoforms are structurally or compositionally related. Indeed the structural analyses required to identify the composition of individual filaments falls outside the scope of this study, however electron microscopy of individual filaments composed of either pure isoforms or the mixes does provide some insight as to how the presence of both isoforms might affect tau filament assembly. As shown previously [16] and here in Figure 8, 3R tau is able to form a range of structures from wide loosely coiled paired helical filaments (Figure 8A) to more tightly coiled straight filaments (Figure 8B) when assembled with heparin. These straight filaments resemble the majority of filaments reported [22] and observed with pure 4R tau reactions (Figures 8C and D). Most interestingly, the assembly reactions of 3R and 4R tau isoform mixtures contained both the loose paired helical filaments observed in 3R reactions (Figure 8E) and the straight filaments found in 4R reactions (Figure 8F). This would appear to confirm the suggestion that the 3R tau and 4R tau do not readily copolymerize. Evidence for this hypothesis might be deduced from the increased proportion of filaments formed in the mixed reaction that contained splayed filament ends with amorphous aggregation (Figure 8E–J). This is in sharp contrast to the filaments formed with single isoform reactions that typically have clean, well defined ends (Figures 8A–D), suggesting that the addition of a second isoform to the assembly reaction interferes with not only nucleation but also elongation.

## Discussion

The ability of individual tau isoforms to differentially regulate microtubule assembly or polymerize into tau filaments *in vitro* has been previously demonstrated [23]–[25], however, *in vivo* the three repeat and four repeat isoforms are found together in nearly equimolar amounts in normal adult humans. This is important as filamentous tau deposits in the majority of tauopathies have predominantly 4R or 3R tau inclusions. Furthermore, FTDP-17 mutations have been described that do not alter the primary sequence of tau or the total tau levels expressed, but rather appear to be pathogenic by simply altering the ratio of 3R and 4R tau found in the brain [9]–[12], often by destabilizing the exon 10 stem loop structure and increasing exon 10 expression [26]. These findings indicate that a balanced tau isoform ratio is necessary for maintaining normal brain functions in humans. Although alterations in the normal tau isoform ratio could negatively impact normal tau function leading to neurodegeneration, a tau isoform ratio imbalance could also affect tau aggregation into neurofibrillary tangles. In fact, *in vitro* studies have shown that 4R tau polymerization is favored over 3R tau polymerization under reducing conditions [16], [17] typically found in cell cytoplasm. These studies suggest that significantly altering the normal tau isoform ratio, particularly shifting the ratio towards 4R tau, as seen in several tauopathies, could lead to enhanced tau aggregation and the development of tau fibrillary pathology observed in these tauopathy patients. These observations led to the hypothesis that altering the normal equimolar tau isoform ratio promotes pro-assembly tau folding, aggregation, and filament formation.

Together the thioflavin S binding, ultracentrifugation and electron microscopy data reported here clearly demonstrate that the extent of tau filament assembly is decreased in reactions containing both 3R and 4R tau isoforms compared to those containing solely 4R tau, and this reduced assembly is independent of the reducing conditions or the expression of amino-terminal inserts via exons two or three. These data also suggest that 3R and 4R tau may not preferentially co-assemble even though both isoforms did aggregate when mixed as demonstrated by ultracentrifugation in Figures 4 and 6. Numerous examples of other amyloidogenic proteins capable of aggregation individually, but showing a reduced rate of aggregation when combined in the same reaction, have been reported including prion proteins from different species [27] and different Aβ peptides [28]. Even when co-incubation of two amyloidogenic proteins does lead to increased fibrillization of both proteins, as in the case of tau and alpha-synuclein, analysis of individual fibrils revealed that the vast majority were homopolymers [29]. Ultracentrifugation data measuring total tau aggregation as shown in Figure 4 substantiated the thioflavin S findings on the tau folding that precedes polymerization, while actual filament formation was confirmed and quantified with electron microscopy in Figure 7. Although not definitive, our electron microscopy data further support the idea that 3R and 4R tau may not prefer to form heteropolymers as suggested by the filament splaying and numerous amorphous filament ends observed in the mixed tau reactions shown in Figure 8. This, in conjunction with the quantitative filament formation presented, suggest that the addition of a second isoform to the tau assembly reaction interferes with both filament nucleation and elongation.

The inhibition of single isoform tau assembly with another isoform is not surprising as the subunits for filament assembly are different. This may be especially true under oxidizing conditions where intermolecular disulfide bonds between 3R and 4R tau may create an additional heterodimer subunit type. However, the data presented here suggested this is not an issue, as the inhibition observed when spiking 4R tau reactions with 3R tau was similar in magnitude, regardless of the reducing conditions used. The dose dependent nature of this inhibition suggests that 3R isoforms may not readily nucleate with 4R tau or incorporate into 4R tau filaments in their preferred configuration. That is, 3R and 4R tau may not be interchangeable subunits and might not co-assemble into hetero-polymers. This could imply that 3R and 4R tau filaments may have different structural morphologies and folding characteristics similar to those reported for wild type and P301L tau, where wild type tau exhibited distinct secondary structures depending on the filament seed type used to induce assembly [30]. If 3R and 4R tau were able to form co-polymers, more compatible tau subunits should have translated into more tau filaments at equilibrium in the spiking reactions, and this was not observed. This indicates 3R and 4R tau might not co-assemble extensively, and this would be true unless these mixed subunits formed filament copolymers that altered the equilibrium constant between monomers and polymers such that it was decreased compared to that observed for the 3R or 4R filaments formed from reactions with pure isoforms. This remains a possibility; and in fact, it is not known if pathogenic tau filaments contain multiple tau isoforms or if it is possible to form single tau filaments containing 3R and 4R tau isoforms.

If the isoforms are not able to co-assemble, the observations from the spiking reactions that did not assemble to the levels observed with 4R tau alone indicate 3R tau may not simply bind and release the 4R tau before additional 4R tau is being incorporated, but rather 3R may be sequestering either 4R tau or the inducer. Binding of 3R to 4R tau monomer or polymer may likely occur and slow 4R tau assembly, but at equilibrium the amount of assembled 4R tau would not decrease unless the 3R binding was irreversible. If this occurred, perhaps the amorphous aggregation on the filament ends of the mixing reactions in Figure 8 represents this irreversible binding. Another possibility is that competition between 3R and 4R tau for heparin binding could lead to sequestering of the inducer. Under this scenario, heparin might only promote filament assembly when all of the isoforms bound were the same. Essentially this would mean that as heparin is concentrating tau locally so that nucleation is accelerated, actual filament assembly would then only be occurring when single tau isoform and heparin complexes are available to establish the seed structure. At the beginning of the reaction, enough single 4R isoform and heparin seeds would be available to promote 4R tau filament assembly; however, as the 4R tau is incorporated into the filaments, the proportion of mixed isoform seeds would increase. Eventually some heparin would not effectively nucleate 4R tau assembly and perhaps even some 3R filaments would form as was suggested by the ultracentrifugation data. This could result in a decrease in heparin available for nucleation and a decrease in total assembly as single 4R isoforms are mixed or spiked with 3R tau. This would presumably be different from inducers that cause conformational changes in tau unless they also simultaneously concentrated the tau proteins. Again, experiments designed to identify the isoform composition of the nuclei and the assembled filaments could indicate how to best capitalize on these findings in either slowing or accelerating tau aggregation therapeutically.

In fact, the results from the mixing experiments with constant tau to heparin ratios suggest that even small proportions of 3R tau may inhibit the assembly of 4R tau. Data from the spiking experiments with constant heparin (Figures 5, 6, 7) or constant tau to heparin ratios (data not shown) demonstrate that small amounts of 3R tau lower the amount of 4R filament assembly compared to tau reactions supplemented with buffer or more 4R tau. Significantly decreased 4R tau assembly in response to small relative amounts of 3R tau may explain why mice and rats typically express small amounts of 3R tau into adulthood [31] and do not typically develop tau pathology. This is also one possible explanation for the differences in neurofibrillary tangle pathology observed in the 8c and htau mouse models that over-express the same human genomic tau transgene but on different mouse tau backgrounds. In both of these models, the genomic human tau transgene displays significantly altered splicing ratios with greater than 90% 3R tau [32] [33]. The presence of the 4R mouse tau isoforms may balance the shift in the human tau gene splicing towards 3R tau, creating more of equimolar ratio in the 8c mice, which do not develop pathological tau lesions [32]; whereas removal of these ratio balancing 4R mouse tau isoforms was observed to cause to neurofibrillary pathology in the htau mice [33]. With the findings from this study, it suggests that the equimolar ratios of tau observed in humans and in other mammals may not only function to ensure proper microtubule dynamics but also to prevent pathogenic filament formation. Together these reports provide compelling evidence that restoring the normal tau isoform balance in 4R tauopathies, perhaps by stabilizing the exon 10 stem loop and reducing 4R tau expression [26], could provide an effective treatment strategy for human tauopathies involving altered isoform ratios. Certainly, it would be interesting to test this hypothesis in some of the 4R mouse tauopathy models by examining the effects of viral 3R tau transduction on the accumulation of tau lesions, neuronal loss and behavioral deficits.

A clear understanding of the assembly and aggregation behavior of reactions containing different tau isoform ratios, similar to those that occur normally and in human diseases, would aid in the development of strategies to intervene in the disease process. The data presented here provides the first evidence that mixing 3R with 4R tau isoforms results in less tau polymerization into filaments such that altering the normal tau isoform ratio towards 4R tau increased tau assembly. Furthermore, the addition of even small amounts of 3R tau inhibited 4R tau assembly by reducing nucleation and perhaps elongation. Moreover, these effects were independent of the reducing conditions of the reactions or the expression of the N-terminal inserts, and they involved a specific interaction between 3R and 4R tau isoforms. They also provide the impetus for examining the effects other modifications like phosphorylation or truncation have on these isoform assembly effects. This is especially important as many tauopathies alter the normal 3R and 4R tau ratios, while in Alzheimer's disease, all six tau isoforms have been observed in filament preparations. This suggests that distinct mechanisms are involved in a disease specific manner in the formation of unique tau aggregates that are defined by their subunit composition, and perhaps, observed in changes filament structure and morphology. Understanding these differences is ultimately useful in manipulating tau accumulation and may be capitalized on, regardless of whether the tau tangles are protective or harmful.
